# Gun-Shot Wound

**Published:** 1828-06

**Authors:** 


### HOSPITAL REPORTS,

*(Principally condensed from various Periodical Publications.)*

### GUN-SHOT WOUND.

*Case of Gun-shot Wound of the Leg, in which five-sixths of the Structure at the Seat of Injury was destroyed, successfully treated, the Limb being preserved.* Reported by JAMES A. WASHINGTON, M.D. House-Surgeon of the PENNSYLVANIA HOSPITAL.

CHARLES ULARY, aged eleven years, was admitted, under Dr. J. R. BARTON, into the Pennsylvania Hospital, on September 1st, 1827, with a recent gun-shot wound of the leg. A fowling-piece, which he had handed across a ditch to its owner, having been accidentally discharged as soon as received, with its muzzle within two feet of him, its contents had passed entirely through his left leg, just above the ankle-joint, the joint happily escaping uninjured. He was brought into the institution before any application had been made to the part, but not until a few hours had elapsed after the accident, as he resided at the distance of four miles from the city. The wound presented the appearance of a large, ragged perforation, passing through the limb horizontally, and from side to side. The orifice on the inner side of the leg was at least three inches in its vertical, and two inches in its horizontal diameter; but the size of the outer orifice was rather less: their margins were irregularly lacerated, as was the whole surface of the wound. The contents of the gun having passed through the limb from so short a distance, a very considerable destruction of the part had taken place, and it was estimated that five-sixths of its structure, at least, had thereby been carried away.

The parts removed consisted principally of more than an inch of the tibia and fibula, their fractured ends, above and below, being that far removed from each other; also of the corresponding portions of the tibialis posticus and the long flexor muscles of the great toe and the small toes, as well as of the fatty matter interposed between them and the tendo Achillis. The skin covering the limb at the seat of injury had, of course, been in a great measure destroyed. The parts that were left in front of the wound consisted of a sound strip of skin about two inches in breadth, and of the muscles which it covered, the peroneous tertius, tibialis anticus, and the long extensors of the great toe and the small toes, which had not been materially injured: behind there remained the tendo Achillis, with another sound strip of skin, but barely sufficient to cover it. The long and the short peroneal muscles, situated on the side of the leg, and whose tendons pass together, behind the malleolus externus, had also escaped without being seriously injured, and were seen crossing about the middle of the external orifice of the wound, the silvery hue of the tendons being still preserved. But what was the most important of all is, that the anterior tibial artery was found uninjured, in consequence of that portion of the periosteum covering the anterior part of the tibia being left, though but barely, and with a film of bone attached to it, here and there.

A consultation having been held between the attending surgeons of the hospital, Drs. Barton, Hewson, and Parrish, they determined, in consequence of the youth of the boy, and of the circulation being still carried on by the anterior tibial artery, as was manifested by its pulsation and the warmth of the foot, to attempt to save the limb. Although, from the lacerated character of the wound, the hemorrhage was rendered much the less profuse, particularly from the large vessels, yet, from the extensive surface that had been exposed, sufficient bleeding had taken place to produce a very decided effect upon the system. When the boy was admitted, his countenance was extremely pale, but reaction had, in a measure, ensued, and his pulse possessed tolerable force, though very quick in its beat.

The immediate application to the wounded surface, which was suggested by Dr. Barton, and had never before been used, as far as we know, consisted of wheat-bran. His limb was placed upon a soft bed of cotton previously spread over with bran, after being protected by oilcloth, in a kind of box, open at one end and above, and with side lids attached to it by hinges, and used for fractured legs in the hospital. The foot, which had before laid upon its outer side, from the very considerable destruction of the continuity of the wounded part, being properly adjusted, the cavity of the wound was filled with the bran, and the limb for a distance above the wound, as well as most of the foot below it, enveloped in the same article. When the character of bran is considered, it will be understood how very well adapted it is to the dressing of a wound of this nature. It serves to absorb the discharge, and is thereby, in part, converted into a very good poultice; while, by the compression thus induced upon the wounded surface, it will prevent any inordinate hemorrhage, at least in a case so favorable to its applicable as the present. For the first five days the bran was only partially removed, and more fresh bran applied; so that the great pain that would have been produced by daily moving the limb to dress it was obviated.

On the sixth day, the boy being lifted towards the foot of the bed, in order that the box might project beyond it, and over a tub placed beneath, the wound was entirely cleansed, by means of a syringe, with warm water, the bran being easily washed away. On this day, the first after the accident that the wound had been fully exposed to view, its surface, which had been killed by the severity of the injury, presented a darkish aspect, owing to the sloughs not yet having separated. Considerable swelling had taken place at the part, in consequence of the inflammation which had supervened. The discharge which had as yet been seen appeared principally to consist of a thin serous exudation of a reddish hue; only a slight trace of pus having up to this time been observed.

By the eighth day, the upper portion of the wound, or that nearest to the knee-joint, had cast off its sloughs, and the exposed granulations presented a very healthy appearance; while the sloughing from the lower portion, or that nearest the foot, had not taken place: this difference being obviously attributable to the diminished circulation in the lower part of the wound.

On the fourteenth day, the wound exhibited a healthy appearance, but the granulations were less florid than previously. On the twenty-fifth day, the cavity of the wound was observed to be considerably diminished by the luxuriance of the granulations, and the contraction that had taken place in the limb; the loss of bony structure allowing it to be thus diminished in length. On the twenty-eighth day, the cavity was still more reduced in size, and the fragments of the tibia had come into contact, from the contraction of the limb above noticed: the upper fragment was spear-pointed, and consisted of compact structure, while the lower fragment presented several points, or a very uneven surface, and consisted of the cellular structure of which the extremities of the long bones are formed. In comparing the legs together, it was found that the injured limb had been shortened rather more than an inch. The upper and lower fragments of the fibula were also found in contact, but, rather less of this bone being removed than of the tibia, it will be perceived that a slight curvature must have taken place at the seat of injury, in order that the fragments of the bones might thus meet. This bend of the limb inwards was, however, but slight. In consequence of this disposition of the parts, the upper fragment of the fibula projected too much outwards, and, from its outer surface being denuded of periosteum, its white appearance rendered it very conspicuous. Although the fragments had now come into contact and jutted against each other, still the foot required support to prevent its falling to one side.

On the 9th of October, five weeks and a half after the accident, the process of exfoliation was, for the first time, observed to have been completed in a part of the exposed surfaces of bone. It has been mentioned that the lower fragment of the tibia consisted of spongy structure, and in a portion of its exposed surface, though the supply of blood to it was of course, from its situation and the nature of the accident, much less abundant than to the more compact structure of the upper fragment, yet exfoliation here first occurred; a circumstance ascribable to the greater vascularity of the spongy structure of the bone.

From this time to the present date, the process of exfoliation has gone on, and is now very nearly completed: bits of bone thus cast off, or loosened from the living structure, have from time to time been removed. The depth to which the surfaces have exfoliated has in no part been great. The formation of osseous matter has also taken place so as to unite the fragments above and below, and for so long a time, and to such a degree, that for a month past no motion at the wound could be perceived.

It was mentioned in the previous part of this paper that a portion of the anterior periosteal covering of the tibia, with small scales of bone attached here and there, had been saved. This remnant of periosteum has been a prolific source of the osseous secretion. There is, correspondent to this portion, a protuberance consisting principally, as indicated by its hardness, of osseous matter, while the parts of the tibia above and below appear to be very nearly in their natural relation to each other as regards direction. The cavity of the wound has for several weeks been filled up, with the exception of a sinus, in which a small probe may be introduced, that is left for the exit of the bits of bone which are from time to time cast off by exfoliation. As this process seems to be almost entirely completed, it is presumed that this sinus will shortly close. The foot and whole limb is somewhat swelled, and, by measurement, the circumference at the wound is twice that of the correspondent portion of the other limb. This difference will be in a great measure diminished after the cure is completed, and he shall have regained full health.

The injured limb is shortened nearly one inch and a half. The curvature of the limb inwards is only slight, but, to overcome this tendency as far as was practicable, an outward splint and roller were, during the progress of the case, used for a time, but are now laid aside. The application of bran is still used alone, with the exception of a roller gently applied over the whole limb, from the toes to the groin, intended to counteract the disposition to œdema, that has been particularly observed since the discontinuance of the pressure used during the application of the outward splint.*

* American Journal of the Medical Sciences.

